# Aquatic organisms modulate the bioreactivity of engineered nanoparticles: focus on biomolecular corona

**DOI:** 10.3389/ftox.2022.933186

**Published:** 2022-08-19

**Authors:** Wei Liu, Isabelle A. M. Worms, Željko Jakšić, Vera I. Slaveykova

**Affiliations:** ^1^ Department F.-A. Forel for Environmental and Aquatic Sciences, Environmental Biogeochemistry and Ecotoxicology, Faculty of Sciences, Earth and Environment Sciences, University of Geneva, Uni Carl Vogt, Geneva, Switzerland; ^2^ Center for Marine Research Rovinj, Institute Ruđer Bošković, Rovinj, Croatia

**Keywords:** Nanotoxicology, nanoparticles, bio-corona, eco-corona, phytoplankton, invertebrates

## Abstract

The increased use of nanoparticle (NP)-enabled materials in everyday-life products have raised concerns about their environmental implications and safety. This motivated the extensive research in nanoecotoxicology showing the possibility that NPs could cause harm to the aquatic organisms if present at high concentrations. By contrast, studies dealing with influence that organisms could exert on the fate and thus effects of NPs are still very rare. Drawing on the existing up-to-date knowledge we critically discuss the formation of biomolecular corona as one of the mechanisms by which organisms exerted control on the NPs fate in the aquatic and biotic environments. We focused the formation of corona by exogeneous and endogenous biomolecules and illustrated the discussion with the specific example of phytoplankton and aquatic invertebrate species. We highlighted the necessity to incorporate the concept of biomolecular corona within more general framework considering the feedback of aquatic organisms and the control they exert in shaping the fate and impact of NPs in the aquatic and biological environment. In our view such broader perspective will contribute to get novel insights into the drivers of environmental transformations of NPs and their mechanisms, which are important in environmental risk assessment.

## Introduction

The increased use of nanoparticle (NP)-enabled materials in multiple products have raised concerns about their environmental consequences and safety. Indeed, the increasing evidences demonstrate that if the NPs are released into the environment, during their production, use or disposal, there is a possibility that they could cause harm to the living organisms if present at high concentrations (Nowack and Bucheli, 2007; Navarro et al., 2008; Auffan et al., 2009; Wiesner et al., 2009; Fabrega et al., 2011; Levard et al., 2012; Bondarenko et al., 2013; Holden et al., 2016; Lead et al., 2018; Grieger et al., 2019; Joonas et al., 2019; Mortimer and Holden, 2019; Kögel et al., 2020; Nguyen et al., 2020; Spurgeon et al., 2020; Sørensen et al., 2020).

The key questions of how the engineered NPs could affect the aquatic organisms and what are the modifying factors guided the research towards development of sound environmental risk assessment and sustainable nanotechnology over past two decades (Nowack et al., 2012; Batley et al., 2013; Collin et al., 2014; Lead et al., 2018; Chen et al., 2019). Indeed, the wealth of information on various NPs and multiple biological models (cells, tissues, organs, and whole organisms) revealed that the reactivity of the NPs towards living organisms is governed by the complex interplay of 1) NP dissolution, 2) organism-dependent cellular uptake, and 3) induction of oxidative stress and consequent cellular damages (Ivask et al., 2014; von Moos and Slaveykova, 2014; Chevallet et al., 2016; Slaveykova et al., 2020). By contrast, studies exploring of how do organisms alter NPs fate in the aquatic environment are still very scarce.

The current state-of-the-art knowledge revealed that phytoplankton could influence the NPs occurrence and fate in the aquatic environment and thus their ultimate impact (Slaveykova, 2022). Various processes were shown to be involved including: 1) uptake of NPs and (intra-) cellular transformation *via* different processes; 2) production of NPs inside the cells and/or on cell surfaces from dissolved metal species; 3) release of various low and high molecular weight biomolecules, which could interact with NPs (e.g., forming a biomolecular corona) (Slaveykova, 2022). No similar conceptual approach has been yet proposed for other aquatic organisms, such as invertebrates. Nevertheless, it is recognized that the interactions of NPs with different biomolecules present in the ambient water and biological environment play a critical role in modulation of the NPs fate, biouptake and toxicity to aquatic organisms in both freshwater and marine environments (Canesi et al., 2016a; Baalousha et al., 2018; Shakiba et al., 2018; Fadare et al., 2019; Fadare et al., 2020; Sikder et al., 2020; Barbero et al., 2021; Liu et al., 2021; Sikder et al., 2021; Wheeler et al., 2021; Baalousha et al., 2022). For example, biomolecules in the aquatic environment can increase or reduce colloidal stability of NPs depending on their quantity and nature, pH and ionic strength: at low ionic strength (e.g., in freshwater) biomolecules can stabilize negatively charged NPs through electrostatic and/or steric interactions, however at high ionic strength (e.g., in marine water) they can reduce NPs stability and favour the aggregation through the formation of cation-biomolecule bridges (Bundschuh et al., 2018). Biomolecular coronas in invertebrate species together with the consequences for the environmental impact of NPs were thoroughly discussed (Canesi et al., 2017). The key role of exogeneous biomolecular corona played in mitigating the NPs toxicity towards living organisms has also been comprehensively reviewed (Natarajan et al., 2021), as well as the environmental dimensions of protein corona (Wheeler et al., 2021).

**FIGURE 1:**
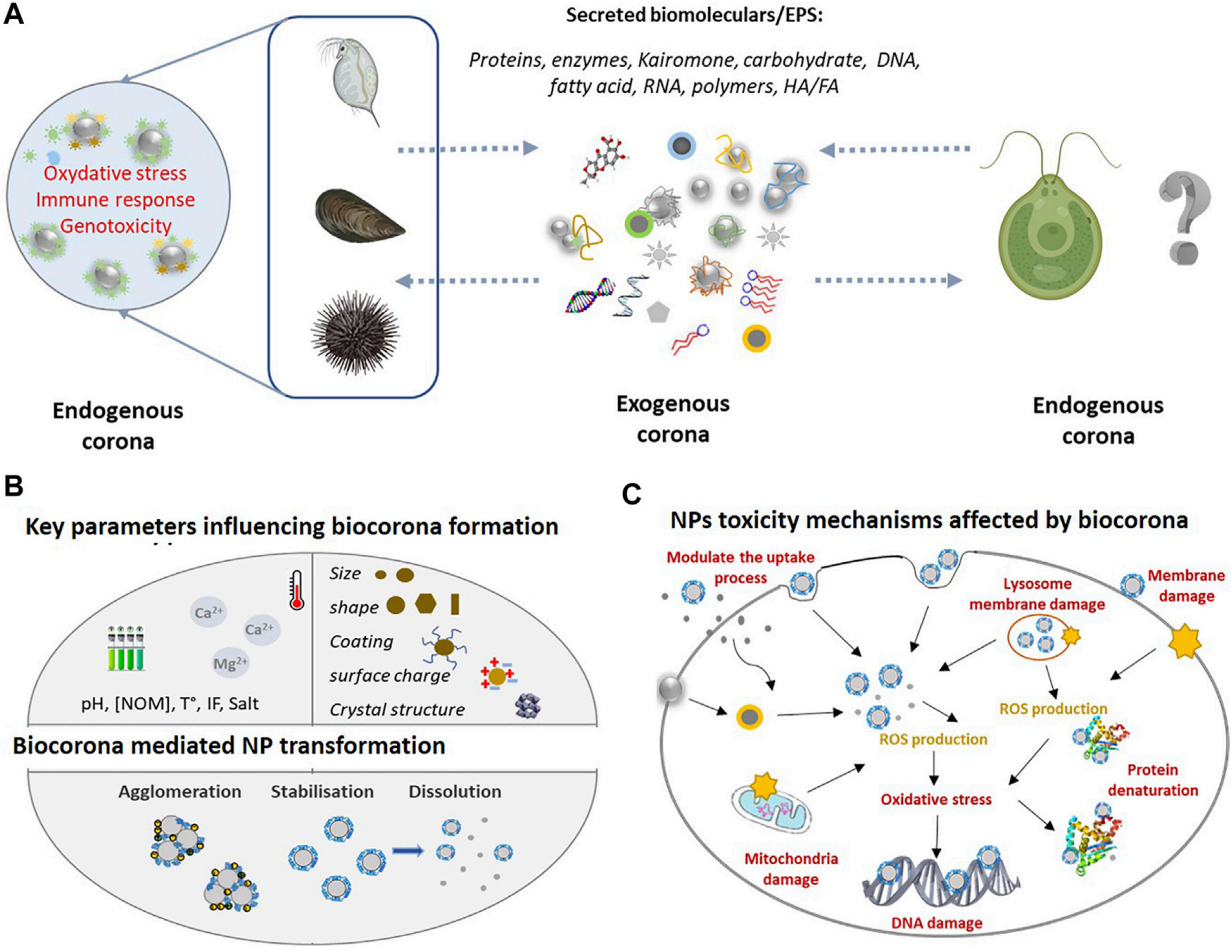
**(A)** Overview of the exogeneous and endogenous corona formation in the case of aquatic phytoplankton and invertebrates in the ambient environment the NPs interact with EPS released by aquatic organisms forming exogenous corona. Once inside the organisms in contact with biological fluids, the NPs corona of exogenous biomolecules will be replaced by endogenous biomolecules which will determine the biological identity and toxicity outcome. Given the high diversity of exogenous biomolecules, the exogenous corona is considered to possess more complex composition as compared with endogenous corona. **(B)** Key environmental variable [e.g., pH, NOM, and temperature (t°)] and NPs intrinsic properties (e.g., size, shape, and coating) influencing corona formation. As a result, the dissolution, stability, and aggregation behaviour of NPs will be affected and thus their bioavailability. **(C)** NPs toxicological mechanisms and their modulation by corona formation. The formation of exogenous corona can affect the adhesion of NPs to the cell surface, alter the interaction between NPs and cell membrane, modulating the uptake process and resulting the membrane damage. Once inside the cell, exogenous corona may mediate the interaction between NPs and cellular components (e.g., mitochondria and nucleus) and biomolecules (e.g., protein and DNA). Exogenous and endogenous corona may affect the extra- and intra-cellular dissolution of NPs, which can induce the reactive oxygen species (ROS) generation and oxidative stress.

Drawing on the existing literature on the formation of biomolecular corona, here we discuss the necessity of broader and integrated approach considering the formation of biomolecular corona as one of the mechanisms by which organisms exerted control on the NPs fate in the aquatic and biotic environments. The discussion of how the aquatic organisms could influence the fate and impact of NPs in aquatic environment will be illustrated with selected examples about the key role of exogeneous and endogenous corona formation in the case of freshwater and marine phytoplankton and invertebrates (Figure 1A).

## Basic principles of biomolecular corona formation

The biomolecular corona is formed by spontaneous adsorption of biomolecules to the highly reactive surface of NPs when they enter in contact with the components of biological or environmental system (Nel et al., 2009; Docter et al., 2015; Moore et al., 2015; Pulido-Reyes et al., 2017; Xu et al., 2020; Wheeler et al., 2021). The scientific understanding on the bimolecular corona formation significantly evolved since the introduction of the term “protein corona” in nano (eco)toxicological research in 2007 through the “eco-corona” and lately “biomolecular corona” encompassing a variety of biomolecules (Wheeler et al., 2021).

The basic principles of formation of biomolecular corona (Lynch and Dawson, 2008; Nel et al., 2009; Tenzer et al., 2011; Monopoli et al., 2012; Walkey and Chan, 2012; Tenzer et al., 2013; Walkey et al., 2014; Docter et al., 2015; Pulido-Reyes et al., 2017; Wheeler et al., 2021) can be summarized briefly as follows:

- The creation of corona is a dynamic adsorption/desorption process where different biomolecules compete for the highly reactive NPs surface; It is accepted that the most abundant biomolecules are adsorbed first, then gradually displaced by the highest affinity biomolecules (Wang et al., 2016). The competition and displacement steps for NPs surface are exhaustively studied for proteins (Hadjidemetriou and Kostarelos, 2017; Wheeler et al., 2021), and also reported for natural organic matter (NOM) (Xu et al., 2020).
- The formation of biomolecular corona on NPs is shown to involve a combination of hydrodynamic, electrodynamic, electrostatic, steric, and bridging interactions. Depending on the characteristics intrinsic for NPs, ambient and biological environments some of the above interactions could prevail (Nel et al., 2009; Pulido-Reyes et al., 2017; Chakraborty et al., 2022).
- Hard and soft coronas are distinguished in the case of proteins. Hard protein corona is characterized by strong binding affinity, long residence time, slow exchange often associated to high conformation changes; while the soft-corona or even protein cloud is formed by loosely bound and rapidly exchangeable biomolecules (Docter et al., 2015). Both of hard and soft corona give the novel bio-identity of the NPs. The most of the available knowledge on the role of the biocorona in NP-cell interactions has been acquired from hard-corona forming proteins, analytically accessible “fraction” (Tenzer et al., 2011; Monopoli et al., 2012; Tenzer et al., 2013; Docter et al., 2015). The role of the soft corona, which presumably determine the new surface charge of the protein-coated NPs and steric interactions of the proteins (García-Álvarez and Vallet-Regí, 2021), and thus the association of NPs with cells, are to further explore. It was also mentioned that the distinction between soft and hard corona seems to mostly “create more confusion than helping to resolve scientific questions” (Docter et al., 2015).
- Biomolecular corona can be formed inside (bio-corona) and outside (eco-corona) of an organism (Pulido-Reyes et al., 2017; Wheeler et al., 2021). It can involve biomolecules released by the organisms or other abiotic material in the aquatic environment (exogeneous corona) and biomolecules found in the biofluids, cytosol etc., (endogenous corona). When NPs are taken up by aquatic organisms, the initial corona of NPs evolves as the exogeneous biomolecules are replaced by endogenous molecules (Xu et al., 2020; Wheeler et al., 2021). The exogenous and endogenous corona are expected to be tightly linked and require more general conceptual consideration.
- Factors affecting the corona formation and corona composition include (Figure 1B): 1) intrinsic NPs properties (such as size, surface coating and functionalization, shape etc.,); 2) ambient medium properties (e.g., ionic strength, pH, presence and characteristics of biomolecules, temperature, light, redox species etc.,); and 3) aquatic organisms [for example, feeding pattern, composition and properties of biological fluids, biomolecules or cell structures (cytoplasm, cell and organelle wall/membrane etc.,)] (Canesi et al., 2017; Fadare et al., 2020; Xu et al., 2020; Wheeler et al., 2021).

The emergence of the above-mentioned principles was based on wealth of the studies mainly with isolated proteins, *in vitro* assays with blood plasma and cell culture media containing serum (Monopoli et al., 2012; Tenzer et al., 2013; Bertoli et al., 2016), *ex vivo* and *in vivo* investigations on tissues and cells (Monopoli et al., 2012; Docter et al., 2015; Durán et al., 2015; Hadjidemetriou and Kostarelos, 2017). Multiplex *ex situ* (involving isolation of the NPs-protein complexes) and *in situ* (no pre-isolation from the ambient medium) approaches used in combination (Monopoli et al., 2012; Docter et al., 2015; Durán et al., 2015) were employed to address the formation and evolution of the coronas, as well as the consequences for NP stability and biological outcome (Docter et al., 2015; Westmeier et al., 2016). Among different NPs, the formation of protein–NP complex was most studied for silver nanoparticles (nAg). The corona formed by the isolated proteins such as bovine or human serum albumin, ubiquitin, yeast extracted proteins etc., (Durán et al., 2015) affected the dissolution, agglomeration and surface charge of the nAg. Further examples include cytochrome (Cyt) C (Kim et al., 2015), some serum proteins (Wimuktiwan et al., 2015) (Burcza et al., 2015) or intracellular metallothionein (Liu et al., 2017). Conversely, the nAg influenced the protein conformation and activity. Interaction of nAg and metallothionein and blood ceruloplasmin resulted in the replacement of native metals of these proteins by Ag^+^ and conformation changes (Liu et al., 2017). Two key antioxidant enzymes: catalase (CAT) and superoxide dismutase (SOD) formed surface complexes with citrate-coated nAg. Consequently, nAg dissolution was promoted together with conformation changes in the CAT, and an impairment of its enzymatic activity. No effect of the nAg–SOD complex on the protein conformation and its enzymatic activity were observed (Liu et al., 2020b). However, firefly luciferase corona on nAg inhibited the enzymatic activity without affecting the protein conformation (Kakinen et al., 2013).

The relationships between nAg synthetic identity (determined by size, surface functionalization, morphology) and the corona formation was demonstrated in simple model systems (Docter et al., 2015; Durán et al., 2015). For example, more pronounced adsorption of bovine serum albumin (BSA) on citrate stabilized 20 nm nAg as compared with 40 and 70 nm nAg was found and resulted in a destruction of part of the helical secondary structure of the BSA (Treuel et al., 2010). For the same size of nAg (20 nm), the affinity of BSA was higher to citrate than to polyvinylpyrrolidone (PVP)-stabilized nAg, an observation explained by prevention of the interactions between nAg surface and S-groups of the proteins by existing PVP corona (Treuel et al., 2012). The nAg morphology (cube, sphere, wire, and triangle) of nAg also influenced the concentration and composition of the proteins in the foetal calf serum and thus their antibacterial activity (Ashkarran et al., 2012). The surface charge of NPs also influenced the protein species to be bound to the surface as a higher charge is expected to induce a more significant binding of proteins to the NPs in comparison with low charged surfaces or no-charge surfaces (Deng et al., 2012). Further recent examples illustrating the formation of corona by model biomolecules, mainly proteins can be found in several comprehensive reviews (Chetwynd and Lynch, 2020; Fadare et al., 2020; Nasser et al., 2020; Barbero et al., 2021; Liu et al., 2021; Natarajan et al., 2021; Wheeler et al., 2021).

Despite such significant advancements in the knowledge concerning the interactions between NPs and biomacromolecules, various issues mainly related with the mechanism of corona formation, its time evolution in complex media, long-term effects and the relevance of corona in the organs where NPs are deposited are still to be resolved (Nel et al., 2009; Monopoli et al., 2012; Tenzer et al., 2013; Miclăuş et al., 2016; Bertrand et al., 2017; Hadjidemetriou and Kostarelos, 2017).

## Biomolecular corona and phytoplankton

### Exogenous corona by biomolecules released by phytoplankton

Phytoplankton release different biomolecules including, extracellular polymeric substances (EPS), which are thought to play a key role in the phycosphere (Seymour et al., 2017; Liu F et al., 2020). EPS are widespread in the aquatic environments (Shou et al., 2018). They are predominant fraction of marine NOM (Corsi et al., 2020) and can represent up to 25% of NOM in freshwaters, particularly during algal blooms (Wilkinson et al., 1997). EPS contain polysaccharides, proteins, lipids, RNA, and DNA, however their composition varied with the species and environmental conditions as reviewed (Naveed et al., 2019).

The EPS produced by marine and freshwater phytoplankton were shown to form biomolecular on different NPs which results in altering NPs colloidal stability and aggregation, dissolution, complexing the released metals, influencing the NPs transformations and favoring formation of the metallic NPs and metal oxide NPs from dissolved metals (Zhou et al., 2016; Zheng et al., 2019; Zhang et al., 2020; Junaid and Wang, 2021; Slaveykova, 2022). Recent reviews have comprehensively summarized the latest advances concerning the role of EPS on NPs fate and effects (Xu et al., 2020; Ly et al., 2021; Slaveykova, 2022).

Below we will give some selected examples in which the formation of corona by EPS was explicitly considered and the possible mechanisms discussed (Table 1). For example, EPS released from green alga *Dunaliella tertiolecta* and in particularly exoproteins with molecular weight range from 20 to 80 kDa, bound to nTiO_2_ stabilizing them in marine waters (Morelli et al., 2018; Corsi et al., 2020). The EPS from green alga *Chlorella pyrenoidosa* adsorbed onto four types of different nTiO_2_ materials with primary size of 5, 10, and 40 nm (anatase) and 25 nm (rutile), the adsorption increased with the specific surface area of the NPs and exhibited a selective adsorption of aromatic EPS components (Gao et al., 2019). EPS from bloom-forming cyanobacteria adsorbed to nZnO *via* electrostatic attraction and surface complexation, thus increasing their stability (Xu and Jiang, 2015). A steric stabilization of sulfide/silica-modified zerovalent iron nanoparticles (nZVI) by organic matter released from green alga *Chlamydomonas reinhardtii* at different growth stages, demonstrated that the algae may play important role in the environmental fate of NPs (Adeleye et al., 2016). EPS produced by cyanobacterium *Synechocystis* sp. efficiently stabilized 20 and 50 nm citrate-coated and lipoic acid-coated nAg, but had only minor effect on the PVP-coated nAg of similar size (Jiménez-Lamana and Slaveykova, 2016), showing the importance of the primary surface coating of NPs. EPS isolated from four marine diatoms *Amphora* sp., *D. tertiolecta*, *Phaeocystis globosa*, and *Thalassiosira pseudonana* decreased the stability of both non-functionalized and functionalized (carboxyl- and amine-) quantum dots (QDs) in artificial seawater with a degradation rate of QDs positively correlated to the protein content of EPS (Zhang et al., 2012). The EPS excreted by green alga *Chlorella* sp. reduced significantly nZnO dissolution (Chen et al., 2012). By contrast, the EPS released by diatom *Isochrysis galbana* increased the dissolution of nCuO and nCu (Adeleye et al., 2014). Similarly, the EPS extracted from cyanobacterial blooms increased the released of Zn ions from nZnO (Xu and Jiang, 2015).

**TABLE 1:** Selected examples to illustrate the interaction between released biomolecules from phytoplankton and NPs.

Organisms	Biomolecules	Nanoparticles	Main findings (NPs colloidal stability, changes of EPS production)	References
*D*. *tertiolecta*	EPS released in culture medium	Aeroxide^®^ P25 nTiO2	Stabilization, 20–80 kDa exoproteins involved, No changes in carbohydrate/protein proportion and quantities over 24 h exposure	Morelli et al. (2018)
*C*. *pyrenoidosa*	Extracted EPS	5, 10, and 40 nm (anatase) and 25 nm (rutile) nTiO_2_	Increase adsorption with specific surface area of NPs	Gao et al. (2019)
Cyanobacterial bloom	Extracted EPS	nZnO	Increase colloidal stability by electrostatic interaction and surface complexation, Increase dissolution	Xu and Jiang (2015)
*Chlorella* sp.	EPS released in culture medium	nZnO	Decrease dissolution	Chen et al. (2012)
*C*. *reinhardtii*	EPS released in culture medium	Sulfide/silica-modified zerovalent iron (nFeSSi)	Decrease in dissolution, ROS production and increase of algal hetero-aggregation, decreasing the toxic effects related to increase production of EPS within time of growth	Adeleye et al. (2016)
*Synechocystis* sp	EPS released in culture medium	20 or 50 nm nAg (PVP; citrate; lipoic acid)	Importance of initial NP coating	Jiménez-Lamana and Slaveykova (2016)
*Amphora* sp., *D. tertiolecta, Phaeocystis globose*, *T*. *pseudonana*	EPS released in culture medium	Coated (carboxyl- and amine-) or uncoated QDs	Increase colloidal stability for citrate- and lipoic acid-nAg/no effect for PVP-nAg, Decrease colloidal stability, Increase dissolution with increase protein/carbohydrate	Zhang et al. (2012)
*I*. *galbana*	EPS released in culture medium	nCu and nCuO	Increase of dissolution	Adeleye et al. (2014)
Cyanobacterial bloom	Extracted EPS, MW fraction	nTiO_2,_ primary particle size of 20 ± 10 nm	Differential adsorption capacities according to MW, Predominance of protein adsorption	Xu et al. (2020a)
*Microcystis aeruginosa*	EPS released in culture medium	nCeO_2_, nCuO and nZnO	Alteration of the produced protein vs. polysaccharide composition	Hou et al. (2017)
*C*. *pyrenoidosa*	EPS released in culture medium	nTiO2	Increase the quantity of released biomolecules	Gao et al. (2020)
*Odontella mobiliensis, Skeletonema grethae, Phaeodactylum tricornutum T. pseudonana*	EPS released in culture medium	nTiO2	Decrease of the quantities of released biomolecules	Chiu et al. (2017)

PVP, Polyvinylpyrrolidone; MW, Molecular weight; ROS, Reactive oxygen species.

The EPS are highly diverse in chemical composition and molecular weight, which makes difficult to characterize in depth their molecular specificity when adsorbing onto NPs (Seviour et al., 2019). High molecular weight (HMW, 1 kDa-0.45 μm) and low MW (LMW, <1 kDa) fractions of cyanobacterial EPS adsorbed on nTiO_2_ in molecular weight- dependent manner, with adsorption capacity decreasing in the order HMW- EPS > Bulk-EPS > LMW-EPS (Xu et al., 2020). Given the large variety of microorganisms, and thus the high diversity of released biomolecules, it is likely that each species wcould modify its external surrounding in a specific way (Ly et al., 2021).

The exposure of phytoplankton species to NPs can modulate the release of EPS, their concentration and composition, thus increasing the complexity of the exogeneous biomolecules composition and potentially formed corona. For example, exposure to nCeO_2_, nCuO, and nZnO affected the quantity and composition (ratio protein vs*.* polysaccharide) of the EPS produced by cyanobacterium *M*. *aeruginosa* (Hou et al., 2017). A significant increase in the release of biomolecules by green alga *C*. *pyrenoidosa* in response to nTiO_2_ was observed (Gao et al., 2020). A decrease of the EPS released by four diatom species *O*. *mobiliensis*, *S*. *grethae*, *P*. *tricornutum*, and *T. pseudonana* upon exposure to nTiO_2_ was reported (Chiu et al., 2017). In addition to the intrinsic chemical identity of the NPs, other NPs properties such as size, surface changes have been shown to affect the release of EPS (Hou et al., 2017; Gao et al., 2020; Junaid and Wang, 2021). The above examples clearly illustrated the existing feedbacks between the NPs exposure and the capacity of phytoplankton species to produce EPS.

In addition, the formation of exogeneous corona in NPs was shown to decrease their bioavailability and toxicity to phytoplankton species (Figure 1C) by three major mechanisms as reviewed (Natarajan et al., 2021): 1) complexation of the metals ions released soluble NPs and decrease of their bioavailability; 2) decreasing the excessive ROS formation, oxidative stress and damage, and 3) altering the attachments of the NPs to the algal cells by modifying the steric and electrostatic interactions between the NPs and cells. It was suggested that the EPS secretion in algae exposed to NPs can be considered as a possible detoxification mechanism (Zhang et al., 2012).

### Endogenous biomolecular corona formation in phytoplankton

In the case of phytoplankton species, no yet studies verified the formation of endogenous corona in the cells. Nevertheless, for the species exhibiting endocytosis, NPs accumulated and transformed into the cell vacuoles as shown for nAg in golden-brown alga *Ochromonas danica,* nAg or nSe in *Poterioochromonas malhamensis* and ferrihydrite NPs in *Euglena intermedia* (Miao et al., 2010; Wang et al., 2013; Liu et al., 2020a; Chen et al., 2022). However, almost nothing is known regarding the biocorona formed during this process. The accumulation of nAg, nAu, nCuO, nTiO_2_, and QDs in the food vacuoles of *Tetrahymena thermophila* by phagocytosis, endocytosis and other unknown mechanism was evidenced (Mortimer et al., 2014a; Mortimer et al., 2014b). After 24 h exposure the micrometre-size heteroaggregates containing nAg or QDs and biomolecules were ejected from the vacuoles, revealing the importance of the interaction of NPs with the endogenous biomolecules inside the cells (Mortimer et al., 2014b). However, no similar studies yet exist for endocytic phytoplankton species.

## Biomolecular corona and invertebrates

### Exogenous corona formation by biomolecules released by invertebrates

Invertebrates, such as crustacean *Daphnia magna*, bivalve *M. galloprovincialis* and sea urchin *Paracentrotus lividus*, are known to release different biomolecules as reviewed (Canesi et al., 2017; Xu et al., 2020; Wheeler et al., 2021). For example, *D. magna* is known to release biomolecules into their surroundings including kairomones, enzymes and proteins from expelled gut, chitin-based carbohydrate from moulting, digestive enzymes and undigested or partially digested matter (Nasser et al., 2020), which are likely to form corona and thus to affect the bioreactivity of NPs*.*

Neonates of *D. magna* were shown to release of proteins, such as Type VI secretion system (74.8 kDa) and Sensor protein QseC (50.4 kDa) (Nasser and Lynch, 2016). These proteins formed exogenous corona on carboxyl- and amine polystyrene NPs (nPS-COOH or nPS-NH_2_) resulting in an enhancement of their uptake and less efficient removal from the gut of *D. magna* (Nasser and Lynch, 2016). By contrast, corona on nAu by proteins secreted from daphnids reduced the nAu aggregation and decreased the toxicity by preventing their surface interaction (Mattsson et al., 2018). An exoprotein corona was formed on nPS-NH_2_ in *D. magna* culture medium and contained proteins responsible for immune defense, cell maintenance, and antipredator response. The size, surface functionalization and aging of NPs were shown as major factors affecting a formation of exogenous corona (Junaid and Wang, 2021).

The existing literature revealed that the exogenous corona formed in the medium surrounding invertebrates is dominated by proteins and confirm its importance as a modulator of NP uptake and toxicity to invertebrates (Nasser et al., 2020). Corona formed by alginate and chitosan on nCeO_2_ did not affect the biouptake, but alginate coated nCeO_2_ induced lipidic peroxidation in marine bivalve *Mytilus galloprovincialis* (Nigro et al., 2021) and altered energetic metabolism and osmoregulation in freshwater bivalve *Dreissena polymorpha* (Della Torre et al., 2021). Interestingly, long-term exposure at low concentrations to nCeO_2_ coated with alginate and chitosan showed significant modulation of electron transport system activity, suggesting a different strategy of defense in response short -term high concentration nCeO_2_ exposure (Nigro et al., 2021). Proteins present in gill mucus of blue mussel *Mytilus edulis* formed a corona on nSiO_2_ and five different types of nTiO_2_ (size and crystal structure); some few proteins in the corona showed a specific recruitment pattern according to the NPs type or crystal structure (Bourgeault et al., 2017). The interactions of NPs with natural bio-molecules, e.g., polysaccharides which and largely present in the marine environment, and impart negative charge to the NPs in particular, could affect NPs toxicity by altering the interactions towards organisms and enhancing NPs biological effects (Nigro et al., 2021).

### Endogenous biomolecular corona formation in invertebrates

Much more extensive is the existing literature considering the formation of the endogenous corona. Endogenous corona determined biological identity of the NPs significantly modulates the cellular uptake, biodistribution of NPs (Treuel et al., 2013) and toxicity outcome in invertebrates (Lynch and Dawson, 2008; Nel et al., 2009; Docter et al., 2015; Durán et al., 2015; Zimmermann et al., 2017; Rastgar et al., 2022). For example the implication of NPs biomolecular corona in the immune response is also emerged in invertebrate models such as *D. magna*, *M. galloprovincialis*, and *P. lividus* (Wheeler et al., 2021). However, the research of biological effects and toxicological consequences of NPs coated with different biomolecules and formed biocorona in marine invertebrates is very limited. In recent years several researches have been provided for Mediterranean mussel *M. galloprovincialis* hemocytes and sea urchin *P*. *lividus* coelomocytes (Table 2).

**TABLE 2:** Selected examples of biological effects and toxicological consequences of nanoparticles coated with different biomolecules and nature of formed biocorona in marine and freshwater invertebrates.

Organism	Biomolecule	Nanoparticles	NPs main findings (aggregation, effect, MoA, toxicity, and consequence)	References
*M*. *galloprovincialis*	hemolymph serum	50.0 nm nPS-HN_2_	180 nm agglomerates formation, interaction with MgC1q6 protein, lysosomal and plasma membrane damage, ROS production, p38 MAPK signalling dysregulation	Canesi et al. (2016a); Canesi et al. (2016b); Canesi et al. (2017)
		9.0 ± 4.0 nm nCeO_2_	interactions with extracellular Cu,Zn-SOD, increased ROS production, activation of O_2_^−^ scavenging activity	Auguste et al. (2021b)
		P25 nTiO_2_	interactions with extracellular Cu,Zn-SOD, no immune response observed	
		50.0 nm nPS-HN_2_ s	Z-average size of about 178.0 ± 2.0 nm, smaller and ticker hemocyte, rich of filopodia and formed lace-like pattern, decrease of lysosomal membrane stability and increased lysozyme activity, decrease of phagocytosis	
		60.0 nm nPS-COOH	Z-average size of about 189.1 ± 48.6 nm, short and long filopodia formation, increased lysozyme release by hemocytes	
		18.3 nm nZnO s	NPs surface energy and its uptake efficiency modulation, NPs lipid peroxidation and immune-related generation of ROS (•O2^-^, NO)	Efthimiou et al. (2021)
	alginate	5.0 nm nCeO_2_	electron transport system activity modulation, lipid peroxidation, GST activity increase and GR activity decrease	Nigro et al. (2021)
	chitosan	5.0 nm nCeO_2_	electron transport system activity modulation, enhanced antioxidant SOD, GPx, and GST activity, GR activity decrease	Nigro et al. (2021)
*P*. *lividus*	coelomic fluid	47.7 ± 1.2 nm nPS-NH_2_ s	Z-average size of about 150 nm, concentration- and time-dependent lysosomal membrane damage and apoptotic-like nuclear alterations in phagocytes	Marques-Santos et al. (2018)
		25.0 ± 3.0 nm PVP-nAu s	concentration-dependent mediation of coelomocytes phagocytosis, inflammation, immunological respons involving TLR4 signalling pathway	Alijagic et al. (2021a)
		25.0 ± 3.0 nm PVP-nAu	expression of CD45 and CD14 antigens on surface of the particular subset of coelomocytes	Alijagic et al. (2021b)
*D*. *magna*		100 nm nPS-HN_2, nPS-COOH_	exogenous corona formation; enhancement of their uptake and less efficient removal from the gut of *D. magna*	Nasser and Lynch (2016)
		25 nm nAu	Exocorona reduced the nAu aggregation and decreased the toxicity by preventing their surface interaction	Mattsson et al. (2018)
*D*. *polymorpha*	alginate	nCeO_2_	energetic metabolism and osmoregulation modulation	Della Torre et al. (2021)
		20.40.100 nm PVP-nAg	accumulation in the soft tissue, the alteration of different morphological and functional characteristics of mussel hemocytes and gill cells, experiencing oxidative stress and the genotoxicity due released Ag	Katsumiti et al. (2015)

GPx, Glutathione peroxidase; GR, Glutathione reductase; GST, Glutathione S-transferase; MAPK, Mitogen-activated protein kinase; MgC1q6 protein, *Mytilus galloprovincialis* putative Complement Component C1q domain; MoA, Mode of action; PS, Polysytene; PVP, Polyvinylpyrrolidone; ROS, Reactive oxygen species; SOD, Superoxyde dismutase.

The interaction of NPs with the mussel biofluids, in particular the haemolymph, allowed to identify biomolecular corona formed with haemolymph serum proteins, and to assess the interaction of biomolecular corona-modified NPs with haemocyte. Protein corona formed in haemolymph serum of *M. galloprovincialis* was dependent on the NPs type and size (Canesi et al., 2016b; Canesi et al., 2017). The positively charged nPS-NH_2_ were covered rapidly with the corona containing *M. galloprovincialis* putative C1q domain containing protein MgClq6 (Canesi et al., 2016b). However, nPS-NH_2_ of primary size 50 nm, 100 nm, and 1 μm aggregated in haemolymph serum in a size dependent manner, which could explain the discrepancy in their bioaccumulation and translocation into *M. galloprovincialis* hemocytes (Sendra et al., 2020). More recently, study of the specific molecular interactions 100 nm nPS-NH_2_ with components in hemolymph serum of *M. galloprovincialis* revealed a formation of corona containing a *M. galloprovincialis* putative putative C1q domain protein MgC1q44 (F0V481) of 23.6 kDa (Auguste et al., 2021a).

nPS-NH_2_ in contact with hemolymph serum induced significant changes in *M. galloprovincialis* hemocytes morphology, formation of filopodia network, decrease of phagocytosis activity and lysosomal membrane stability and increase of lysozyme activity. nPS-COOH induced short and long filopoda formation, as well as increased lysozyme release by hemocytes. By contrast, the exposure of *M. galloprovincialis* hemocytes to custom made polyvinylpyrrolidone coated gold nanoparticles (PVP-nAu) in artificial sea water doesn’t induce any changes in morphology, phagocytosis activity, lysosomal membrane stability and extracellular ROS production (Auguste et al., 2021b). Cu, Zn—containing superoxide dismutase (Cu,Zn-SOD) prevailed in nCeO_2_ and nTiO_2_ corona (Canesi et al., 2017).

The formation of different type of biomolecular corona resulted in an increase or decrease in the immunotoxicity of either type of NPs for haemocytes. For example, the formation of MgClq6 corona on the nPS-NH_2_ resulted in an increase of ROS generation, lysosomal and plasma membrane damage and dysregulation of p38 mitogen-activated protein kinase (P38MARK) signalling in *M. galloprovincialis* in comparison with the same NPs in artificial sea water (Canesi et al., 2016b). However, the formation of a Cu,Zn-SOD corona on nCeO_2_ activated O_2_^−^ scavenging activity in mussel haemocytes and thus contributed to a mitigation of ROS production. Under the same conditions Cu,Zn-SOD corona did not affected the response of haemocytes on nTiO_2_ (Canesi et al., 2017).

The biomolecular corona formation can be also influenced by the NPs form and shape. The nCeO_2_ with negative charge and the rounded shape formed Cu,Zn-SOD biomolecular corona in hemolymph serum which triggered higher changes in the immunological parameters such as ROS and phagocytosis capacity. Thereby, it was observed that difference in surface *ζ* potential play an important role in NPs association with particular extracellular proteins: *M. galloprovincialis* putative C1q domain containing protein (MgC1q6) with nPS-NH_2_ s, and Cu,Zn-SOD protein with the nTiO_2_ and nCeO_2_ with negative *ζ* potential (Canesi et al., 2017). However, faceted shape nCeO_2_ with neutral surface charge did not show either biocorona formation in hemolymph serum or significant immunological parameters responses (Sendra et al., 2018). In the freshwater mussel *D. polymorpha,* the only known study demonstrated a rapid accumulation of PVP-coated nAg in the soft tissue and the alteration of different morphological and functional characteristics of mussel hemocytes and gill cells, experiencing oxidative stress and the genotoxicity due released Ag (Katsumiti et al., 2015).

Furthermore, the intralysosomal accumulation of nAg in mussel digestive gland was recently evidenced, with accumulation rate depending on the NPs surface coating and size (Jimeno-Romero et al., 2017). The induction of inflammation effect in the gills of mussels exposed to nAg related directly to nanoparticle size and exposure time (Bouallegui et al., 2017). More recently custom made, nZnO promote lipid peroxidation and immune-related generation of ROS, like superoxide anion (^•^O_2_^−^) and nitric oxide (NO) in hemocytes of *M. galloprovincialis* presumably as a result of protein biomolecular corona modulating the NPs surface energy and uptake efficiency (Efthimiou et al., 2021).

Formation of endogenous corona on the 150 nm nPS-NH_2_ dominated by 180 kDa Toposome precursor was shown in the coelomic fluid of purple sea urchin *P. lividus* (Marques-Santos et al., 2018). Under these conditions a concentration- and time-dependent lysosomal membrane damage and apoptotic-like nuclear alterations in *P. lividus* phagocytic coelomocytes were observed. This finding agrees with previous studies on mammalian cell lines and *M. galloprovincialis* hemocytes and further support the idea of ubiquitous “proton-sponge” effect (leading to lysosomal damage and the induction of cytotoxicity by cationic nanoparticles (Nel et al., 2009).

The complexity of the corona composition was further demonstrated in *P. lividus* coelomic fluid exposed to 50 nm nPS-NH_2_ and nPS-COOH by identifying 13 unique proteins on the hard coronas of both nPS: nectin variant 2 precursor, fitolitin-1, actine related protein 1, and guanine nucleotide binding protein *β* subunit were distinctive for nPS-NH_2_ corona, the fascin, actin cytoskeletal 2A, actin cytoskeletal 3B, and muscle actin isoform X1 were features of the nPS-COOH corona, whereas toposome, nectin precursor, actine cytoskeletal 2B and actin 15B were identified in both modified NPs hard biocorona (Grassi et al., 2019). Furthermore, nTiO_2_ exposed to cell-free and cell-conditioned media develop distinctive corona of predominantly negatively charged proteins involved in cellular adhesion (toposome, galectin, nectin), and cytoskeletal organization (actin, tubulin). nTiO_2_ bound on the outer surface of the cells and within well-organized vesicles, no harmful effects (e.g., oxidative stress and caspase activation) were observed (Alijagic et al., 2019). However, the PVP-nAu in contact with *P. lividus* coelomic fluid acquire biocorona based on three major proteins of about 40, 100, and 170 kDa. A selective biological affinity towards particular proteins (100 kDa proteins in nTiO_2_ versus 40 kDa proteins in PVP-nAu biocorona) in contact with sea urchin coelomic fluid modulated recognition of the NPs by the immune cells and their interactions (Alijagic et al., 2021a; Alijagic et al., 2021b). In addition, it is emphasized that PVP-Au NPs may stimulate a subset of *P. lividus* celomocytes to express on their surface CD45 and CD14 antigens, known to be involved in immune cell maturation and macrophage activation in humans (Alijagic et al., 2021a).

## Discussion and research gaps

The existing state-of-the-art knowledge revealed that the aquatic phytoplankton and invertebrates could exert an important influence on the behavior and thus toxicological effects of the NPs, in particularly *via* formation of the biomolecular corona with the different endogenous and exogenous biomolecules. Exogeneous biomolecules released from phytoplankton were shown to form a corona on NPs and alter their dissolution and stability. The exoprotein fraction of the EPS was suggested to be main components of the exogeneous corona (Wheeler et al., 2021). Much less studies explore the formation of exogeneous corona with biomolecules released by marine and freshwater invertebrates and could be a topic of future research.

The review of extensive literature on the endogenous corona formation in invertebrates demonstrated that the majority of NPs biomolecular corona studies were preformed following *in vitro* exposure in the haemolymph serum of marine bivalve, in a high NPs concentration range, highlighting the crucial role of biomolecular corona formation in their bioavailability and resulting biological response. Different proteins were shown to be involved in the formation of stable corona according to NPs intrinsic properties and biofluid characteristic. The results obtained so far underline the need to understand how NPs transform in complex environmental matrices *in vivo*, and how NPs interacts with biomolecules present in both environmental media and biological fluids. Moreover, given the large diversity of phyla and species, the data on formation of NPs-protein biomolecular corona in aquatic invertebrates remains scarce, in particular in freshwater invertebrates. The formation of endogenous corona in phytoplankton cells need to be further explored and demonstrated, as biocorona could be expected to be an important modulator of the intracellular handling and effects of NPs.

Regardless of the existing differences in the nature, composition, and molecular weights of the biomolecules produced by various aquatic organisms, the dissimilarity in the NPs uptake patterns, the processes controlling the formation of biomolecular corona and modifying factors are similar. Nevertheless, further mechanistic studies by exhaustive identification and quantitative measurements of various LMW and HMW biomolecules which interact with and form exo- and endogenous corona on NPs are highly sought. Gaps in knowledge concerning the mechanism of formation, stability and evolution of biomolecular corona, exchange between exogeneous and endogenous corona components, as well as toxicity outcome in aquatic organisms exposed to NPs, remain to be fully elucidated. Furthermore, a consideration should be given not only on polysaccharides and proteins as main constituents of exogenous and endogenous biomolecular corona, but on different metabolites which are involved in signaling cascades and/or molecular responses to NPs exposure. Indeed, the concept of exometabolite corona has emerged (Chetwynd and Lynch, 2020), and the interplay between exometabolites, important constituents of the phycosphere (Seymour et al., 2017; Liu F et al., 2020), and EPS and the consequences for biocorona formation are still to decipher. With this respect studies combining proteomics and metabolomics approaches could open a novel avenue towards improved understanding of the complexity of biomolecular corona dynamics.

Overall, the present contribution put the concept of biomolecular corona formation in a new perspective and propose its incorporation within more general framework considering the role of aquatic organisms in shaping the fate and effects of NPs in the aquatic and biological environment.
